# Supplementary figures and images for: Relationship between circadian genes and memory impairment caused by sleep deprivation
Source: PeerJ. 2022 Mar 21;10:e13165. doi: 10.7717/peerj.13165 (PMC8944342; doi:10.7717/peerj.13165)

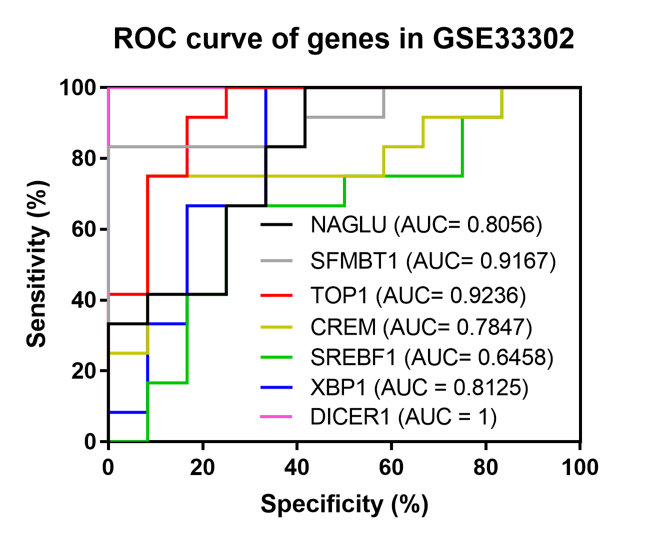

Supplement: Supplemental Information 4 [file peerj-10-13165-s004.png]

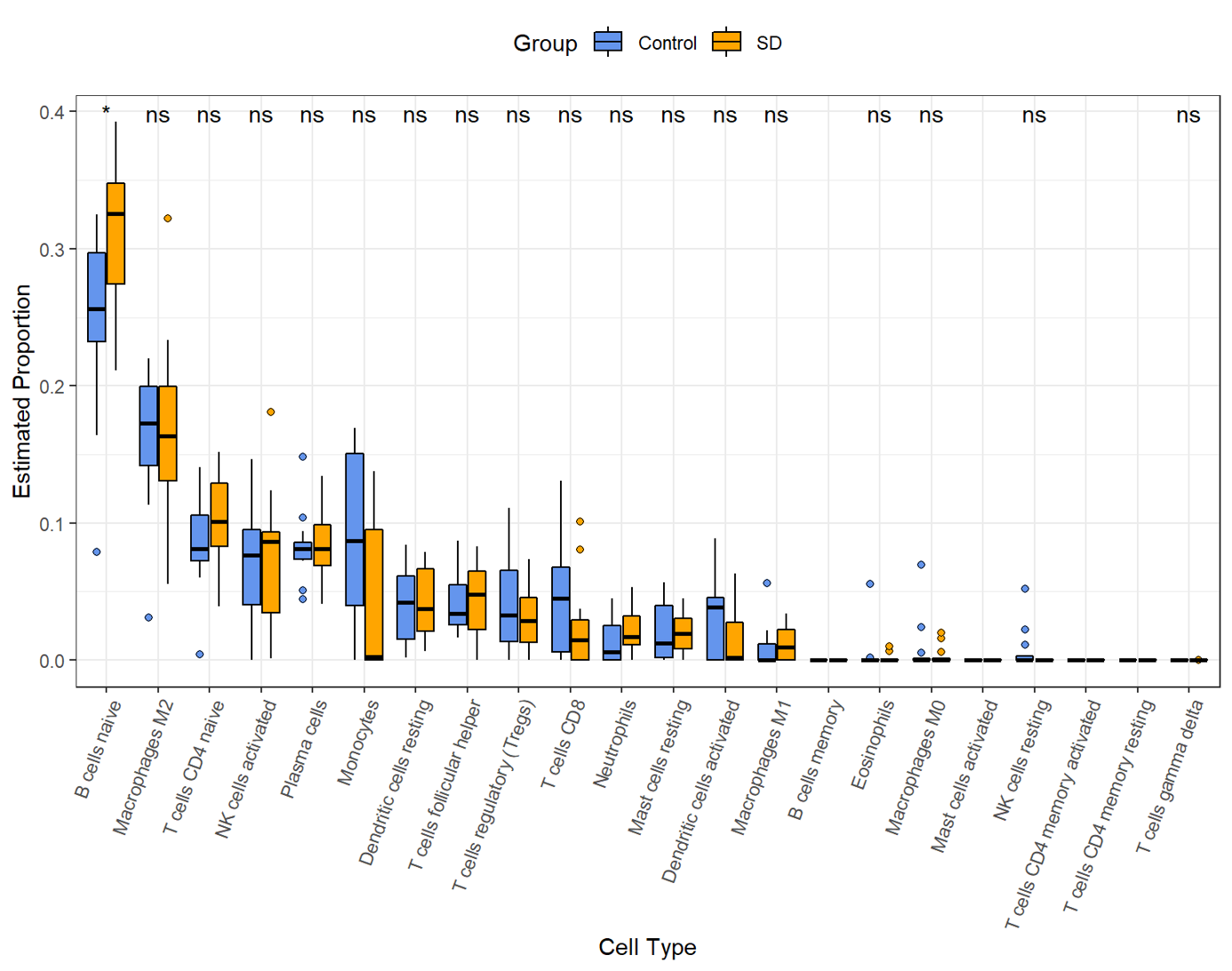

Supplement: Supplemental Information 7 [file peerj-10-13165-s007.png]
